# Regorafenib in Recurrent Glioblastoma Patients: A Large and Monocentric Real-Life Study

**DOI:** 10.3390/cancers13184731

**Published:** 2021-09-21

**Authors:** Giuseppe Lombardi, Mario Caccese, Marta Padovan, Giulia Cerretti, Giovanna Pintacuda, Renzo Manara, Francesca Di Sarra, Vittorina Zagonel

**Affiliations:** 1 Department of Oncology, Oncology 1, Veneto Institute of Oncology IOV-IRCCS, via Gattamelata 64, 35128 Padua, Italy; mario.caccese@iov.veneto.it (M.C.); marta.padovan@iov.veneto.it (M.P.); giulia.cerretti@iov.veneto.it (G.C.); vittorina.zagonel@iov.veneto.it (V.Z.); 2 Clinical and Experimental Oncology and Immunology PhD Program, Department of Surgery, Oncology and Gastroenterology, University of Padua, 35128 Padua, Italy; 3 Radiology Unit, Veneto Institute of Oncology IOV-IRCCS, via Gattamelata 64, 35128 Padua, Italy; giovanna.pintacuda@iov.veneto.it; 4 Department of Neurosciences, Neuroradiology Unit, University of Padua, 35128 Padua, Italy; renzo.manara@unipd.it; 5 Pharmacy Unit, Veneto Institute of Oncology IOV-IRCCS, via Gattamelata 64, 35128 Padua, Italy; francesca.disarra@iov.veneto.it

**Keywords:** regorafenib, glioblastoma, brain tumors, glioma, targeted therapy

## Abstract

**Simple Summary:**

In the last years, treatments for recurrent glioblastoma patients have shown limited efficacy in terms of OS. In the REGOMA trial, regorafenib demonstrated encouraging results in this setting of population. Indeed, in this randomized, phase 2 study the OS was significantly improved in the regorafenib arm compared with the standard lomustine treatment. Noteworthy, based on the REGOMA trial results, regorafenib was included in the NCCN 2021 guidelines as a preferred regimen for recurrent glioblastoma patients. To date, no studies have analyzed the impact of regorafenib in patients treated outside of clinical trials. We performed a large study in order to evaluate the safety and efficacy of regorafenib in the real-life population of recurrent GBM patients. Our results were superimposable to the ones reported in the REGOMA trial and regorafenib should be considered as an interesting drug in a population with a very poor prognosis and an unmet clinical need.

**Abstract:**

Despite multimodal treatment with surgery and radiochemotherapy, the prognosis of glioblastoma remains poor, and practically all glioblastomas relapse. To date, no standard treatment exists for recurrent glioblastoma patients and traditional therapies have showed limited efficacy. Regorafenib is an oral multi-targeted tyrosine kinase inhibitor showing encouraging benefits in recurrent GBM patients enrolled in the REGOMA trial. We performed a large study to investigate clinical outcomes and the safety of regorafenib in a real-life population of recurrent glioblastoma patients. Patients receiving regorafenib outside clinical trials at the Veneto Institute of Oncology were retrospectively reviewed. The major inclusion criteria were: histologically confirmed diagnosis of glioblastoma, prior first line therapy according to “Stupp protocol”, Eastern Cooperative Oncology Group (ECOG) performance status score ≤1. According to the original schedule, patients received regorafenib 160 mg once daily for the first 3 weeks of each 4-week cycle. The primary endpoints of the study were overall survival and safety. A total of 54 consecutive patients were enrolled. The median age was 56, MGMT methylated status was found in 28 out of 53 available patients (52.8%), *IDH* mutation in 5 (9.3%) and 22 patients were receiving steroids at baseline. The median overall survival was 10.2 months (95% CI, 6.4–13.9), the OS-12 was 43%. Age, MGMT methylation status and steroid use at baseline were not statistically significant on a multivariate analysis for OS. Patients reporting a disease control as best response to regorafenib demonstrated a significant longer survival (24.8 months vs. 6.2 months for patients with progressive disease, *p* = 0.0001). Grade 3 drug-related adverse events occurred in 10 patients (18%); 1 patient (2%) reported a grade 4 adverse event (rash maculo-papular). No death was considered to be drug-related. This study reported the first large “real-life” experience of regorafenib in recurrent glioblastoma. Overall, our results are close to the ones reported in the previous phase 2 study, despite the fact that we had a longer survival. We showed the encouraging activity and tolerability of this treatment in recurrent glioblastoma patients when used as a second-line treatment.

## 1. Introduction

Glioblastoma is the most common primary malignant brain tumor in the adult population [1], with a poor prognosis and limited therapeutic alternatives. The standard of care in newly diagnosed glioblastoma is maximally safety resection and subsequent concomitant chemoradiotherapy with temozolomide [2]. Despite this type of treatment, a recurrence of the disease occurs in almost all patients after 6–9 months following primary therapy [3,4]. Treatment for disease recurrence remains a challenge [5,6,7], and enrollment in clinical trials should be recommended; indeed, second surgery, re-irradiation and traditional chemotherapy with nitrosoureas, or temozolomide rechallenge or bevacizumab demonstrated limited efficacy [8,9]. Immunotherapy with checkpoint inhibitors, such as nivolumab or pembrolizumab, showed poor results [10,11].

Regorafenib is an orally multikinase inhibitor against several targets such as *VEGFR1-3*, *TIE2*, *KIT*, *RET*, *RAF1*, *BRAF, PDGFR* and *FGFR,* and is approved as monotherapy for the treatment of hepatocellular carcinoma, gastrointestinal stromal tumors and colorectal cancer [12,13,14]. This drug has also demonstrated a significant reduction of gadolinium extravasation in rat glioblastoma tumor xenograft in preclinical studies, with antitumor activity and inhibition of tumor growth through the reduction of vascularization and the inhibition of the PDGFR pathway [15,16].

In 2019, Lombardi et al., published the results of the REGOMA trial [17], in which regorafenib was tested in recurrent glioblastoma patients compared to lomustine. In this phase II randomized controlled multicenter study, 59 and 60 recurrent glioblastoma patients were treated with regorafenib and lomustine, respectively. The overall survival was significantly improved in the regorafenib arm compared with the lomustine (HR 0.50, 95% CI 0.33–0.75, *p* = 0.0009). A major activity for regorafenib was seen also in terms of neuroradiological assessment (according to RANO criteria) and progression-free survival. Based on these results, regorafenib was included in the NCCN 2021 guidelines as a preferred regimen for recurrent glioblastoma patients, and the Italian Agency of Medicine (AIFA) also approved its use for Italian patients in October 2019.

We performed this large and monocentric study in order to evaluate the safety and efficacy of regorafenib in the real-life population of recurrent GBM patients.

## 2. Materials and Methods

We performed a monocentric, retrospective study analyzing recurrent glioblastoma patients treated with regorafenib at our oncological center. The enrollment criteria were similar to the ones reported in the REGOMA trial. The inclusion criteria were: histologically confirmed diagnosis of glioblastoma, prior first line therapy according to the “Stupp protocol”, age >18 years, Eastern Cooperative Oncology Group (ECOG) performance status score ≤1, adequate bone marrow, liver and renal function. The patients who underwent surgery at the time of the first tumor progression were considered eligible only if they had histological confirmation of glioblastoma recurrence. The exclusion criteria were: prior therapy with regorafenib or other antiangiogenic drugs, two or more lines of chemotherapy, uncontrolled hypertension, myocardial infarction, arterial thrombotic or embolic events or pulmonary embolism within six months before regorafenib treatment, active or chronic hepatitis B or C virus infection requiring antiviral therapy and use of strong cytochrome P3A4 (CYP3A4) inhibitors or inducers. 

The clinical, radiological and molecular data of all patients were collected prospectively.

The enrolled patients received regorafenib 160 mg (four 40 mg tablets) orally once daily for the first 3 weeks of each 4-week cycle. The treatment was continued until disease progression, death, unacceptable toxicity or consent withdrawal. According to the REGOMA trial, regorafenib dose reduction (120 mg than 80 mg) was allowed based on the degree of related toxicities reported and the dose could be re-escalated at discretion of the investigators once the toxicity resolved to baseline levels. Clinical, serum chemistry and haematological evaluations were performed every two weeks for the first two cycles and every four weeks thereafter. Radiological assessment was done with gadolinium brain MRI about every 8–12 weeks from the first drug administration until disease progression; the response was evaluated according RANO criteria. Adverse events were graded according to the National Cancer Institute Common Terminology Criteria for Adverse Events v5.0 [18].

The primary endpoint was the overall survival defined as the time from the start of the treatment to death, occurring due to any cause. The secondary endpoints were progression free survival defined as the time from the start of regorafenib to disease progression according to RANO criteria or death, the proportion of patients achieving Disease Control (DC) (defined as stable disease, partial response and complete response according to RANO criteria), Objective Response Rate (ORR) (defined as partial and complete responses according to RANO criteria) and safety. The overall survival and the progression free survival was estimated using the Kaplan-Meier methods. For the primary endpoint of the overall survival, patients who were still alive were censored from the date of analysis; Patients without progression were censored at their final follow-up visit. The Log-rank test was used for the univariate analysis, the Cox Proportional hazards regression model was used for the multivariate analysis. A multivariate Cox proportional hazards regression model was used for multivariate analysis to test the effect of prognostic factors in terms of OS and PFS. In order to assure that all pertinent and potentially predictive variables are studied, a univariate inclusion criterion of *p* ≤ 0.2 was used for the multivariate model. Categorical variables were compared using Fisher’s exact test. *P* values were based on 2-side testing and differences with a *p* ≤ 0.05 were considered significant. Adverse events and laboratory abnormalities were reported by the worst grade experienced by the patient.

Statistical analyses were done with SPSS software (version 26). 

Ethics approval was obtained by the Ethics Committee of the Veneto Institute of Oncology, IOV-IRCCS (EC number: 2020/154). Written informed consent was required from all patients involved in this study.

## 3. Results

**Figure 1:**
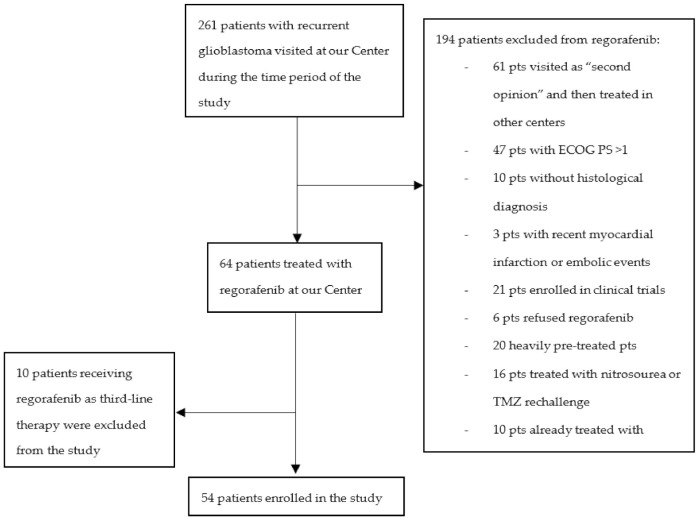
Flow-chart of the study.

We enrolled 54 patients treated with regorafenib at the Veneto Institute of Oncology from February 2018 to September 2020 (see Figure 1).

Median age was 55 (30–77). *MGMT* was methylated in 28 out of 53 available patients (52.8%); *IDH1/2* mutation was found in 5/54 patients (9.3%). Second surgery at the time of the first relapse was performed in 16/54 patients (29.6%), and 22/54 (40.7%) patients were receiving corticosteroids at the start of regorafenib treatment. Among all treated patients, 35/54 (65%) received a subsequent cancer therapy as third-line therapy after regorafenib (patients characteristics were summarized in Table 1). 

At the analysis cutoff date of 18 January 2021, the median follow-up was 11.1 months. Thirty (55.5%) out of 54 patients treated had died, and 50 (92.6%) out of 54 patients had discontinued treatment. The most common reason for treatment discontinuation was disease progression in 47/50 (94%) of patients.

**Figure 2:**
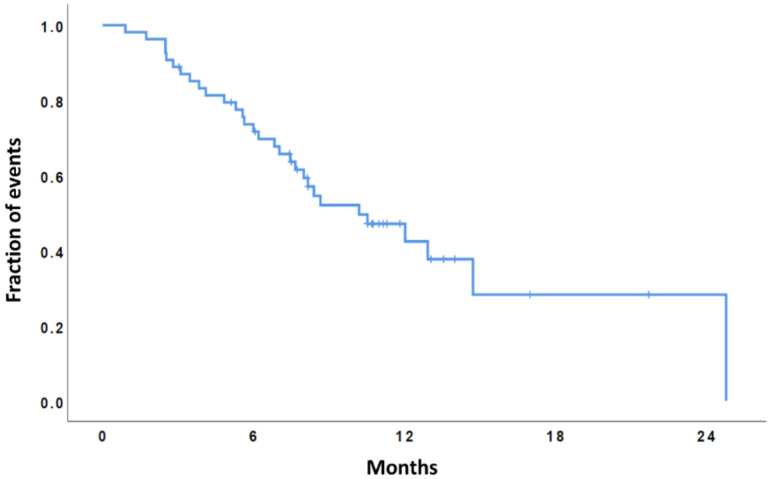
Kaplan-Meier curve of overall survival (the median OS was 10.2 months, 95% CI 6.4–13.9).

**Figure 3:**
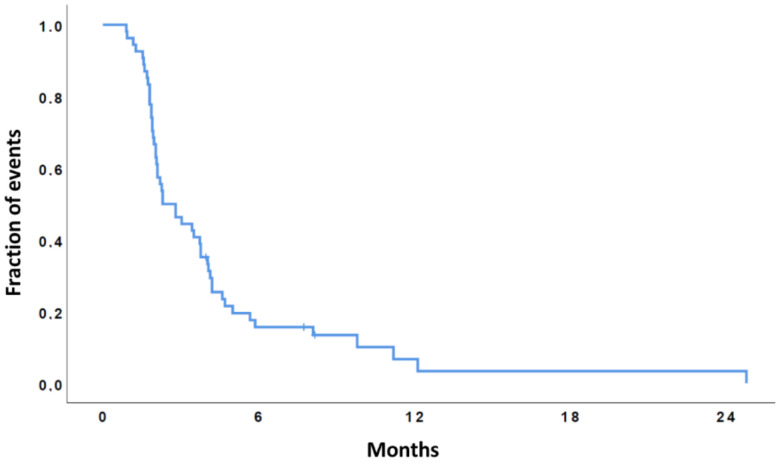
Kaplan-Meier curve of progression free survival (the median PFS was 2.3 months, 95% CI, 1.3–3.3).

The median overall survival (OS) from starting regorafenib was 10.2 months (95% CI, 6.4–13.9), while the OS-12 rate was 43%. The median PFS was 2.3 months (95% CI, 1.3–3.3), and the 6-months PFS rate was 18% (see Figure 2 and Figure 3).

**Figure 4:**
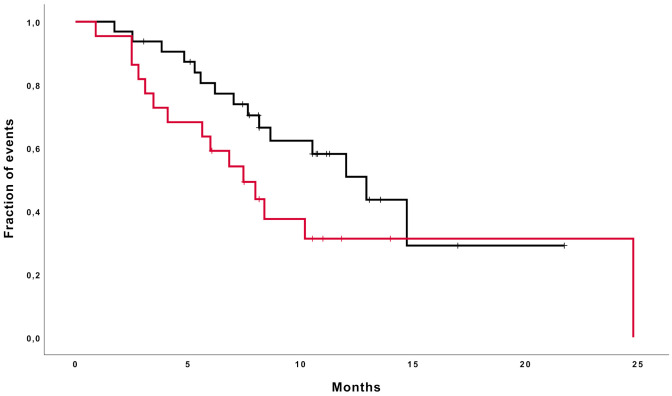
Median overall survival curves in patients taking (red line) or not taking (black line) steroids at baseline (7.4 months and 12.9 months, respectively; *p* = 0.09).

Age, second surgery, IDH mutational status, MGMT methylation status, corticosteroids use at baseline (see Figure 4) and the subsequent treatment after regorafenib were not correlated to a significant impact on the overall survival on the univariate analysis (see Table 2).

On the multivariate analysis for the overall survival, age, MGMT status and corticosteroid use at the start of regorafenib treatment remained non-statistically significant (see Table 3).

All patients were evaluable for response: four patients (7.4%) reported a partial response, 21 (38.8%) patients reported a stable disease. The disease control rate was demonstrated in 25 (46.3%) patients. No patient showed a complete response to treatment. (see Table 4). Noteworthy, only four patients (18%) discontinued or reduced corticosteroids while taking regorafenib.

**Figure 5:**
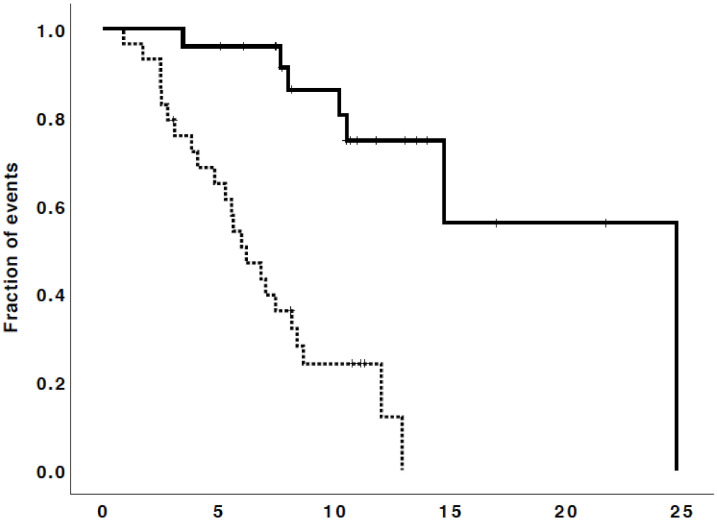
Survival curves of the patients reporting a disease control (continuous line, median OS of 24.8 months) and the patients with progressive disease (dashed line, median OS of 6.2 months) (*p* = 0.0001).

It is worth noting that patients reporting a disease control showed a statistically longer OS of 24.8 months (95% CI not available) compared to patients with progressive disease with a median OS of 6.2 months (95% CI, 4.5–7.8) (*p* = 0.0001) (see Figure 5).

Drug-related adverse events led to a dose reduction of regorafenib in 20/54 (37%) patients, while in three patients (6%) it was permanently discontinued. In the entire study population, 49/54 (90.7%) of patients developed at least one treatment-related adverse event but for almost all cases, these were drug-related grade 1–2 adverse events. The most frequent grade 3 adverse events were hand-foot skin reaction (four patients), fatigue (one patient), mucositis (one patient), hypertranaminasaemia (two patients), increased lipase and amylase (one patient) and skin rash (three patients); only one (one patient) grade 4 adverse event was recorded (skin rash). No deaths were considered drug-related. (Table 5)

## 4. Discussion

In this retrospective study, we reported the activity, efficacy and safety analysis of regorafenib treatment in patients with relapsed glioblastoma who had received chemoradiation therapy as first line treatment. We showed results similar to the ones reported in the Regoma trial [17] We reported a median OS of 10.2 months (95% CI 6.4–13.9) and the OS-12 rate of 43%; albeit with the limitations and bias of a retrospective study, the data are particularly encouraging. The median PFS and the 6 m-PFS rate reported in our study were very similar to the ones showed in the REGOMA trial; indeed, we reported a median PFS of 2.3 months (95% CI, 1.3–3.3) versus 2.0 months (95% CI, 1.9–3.6) of the REGOMA trial and a 6 m-PFS rate of 18% compared to 16.9% in the REGOMA. However, the neuroradiological response to regorafenib was very close to the data presented in the prior study by Lombardi G et al.; in fact, as reported in Table 4, 46.3% of patients treated with regorafenib achieved a disease control (44% in Regoma trial) and among these, 38.9% achieved a stable disease (39% in the REGOMA) and 7.4% a partial response (3% in the REGOMA); it is worth noting that no complete response was demonstrated (versus 2% in the Regoma trial).

Although we reported a longer overall survival compared to other prior studies analyzing bevacizumab in recurrent glioblastoma (medial OS was 7.2 months in Kreisl et al. and 9.2 months in Friedman et al.) [19,20], our results in terms of median PFS and 6 m-PFS were inferior (median PFS was 3.7 months in Kreisl et al. and 4.2 months in Friedman et al.; 6 m-PFS were 29% in Kreisl et al. and 43% in Friedman et al.). On the other hand, in the recent phase 3 study (CheckMate 143) [11], the patients treated with bevacizumab showed a median PFS of 1.5 months and a median overall survival of 10.0 months. Yet, the AVAREG trial, a phase 2, randomized and non-comparative study of fotemustine or bevacizumab in recurrent glioblastoma patients, showed a median OS of 7.3 months and 8.7 months in patients treated with fotemustine and bevacizumab, respectively [21]. 

On the other hand, Desjardins et al. investigated survival and safety of 74 patients with recurrent glioblastoma treated with bevacizumab-containing regimens in clinical practice; this retrospective study showed a median OS and PFS from bevacizumab initiation of 11.1 and 6.4 months, respectively [22].

Moreover, other recent treatments showed interesting results in this setting of patients with a median OS longer than one year; indeed, in a phase 1 study, recurrent high-grade glioma patients treated with TOCA-511 (Vocimagene Amiretrorepvec) showed a median OS of 13.6 months [23]; however, in a subsequent phase III study this treatment did not improve overall survival compared to standard of care (11.10 months versus 12.22 months, respectively) among patients with first or second recurrence of GBM or anaplastic astrocytoma [24]. In other recent study, Desjardins et al. conducted a dose-finding and toxicity study in recurrent GBM patients treated with intratumoral infusion of the recombinant nonpathogenic polio-rhinovirus chimera (PVSRIPO); in this study, 61 patients were enrolled showing an OS of 12.5 months and the OS-24 rate of 21% [25].

Overall, based on these results, regorafenib should be considered as an interesting drug in a population with a very poor prognosis and an unmet clinical need. 

However, we showed that patients reporting a disease control to regorafenib treatment can have a longer survival compared to patients with a progressive disease, confirming the important role of regorafenib for these patients in terms of efficacy (see Figure 5). Interestingly, only four patients (18%) versus 55–58% of patients treated with bevacizumab [19,22] were able to decrease their requirement for corticosteroids: this may demonstrate the lower anti-edema effect of regorafenib compared to bevacizumab. 

Unfortunately, we did not perform an exploratory analysis to study some predictors of efficacy; indeed, two prior studies have investigated the role of molecular predictors of regorafenib efficacy in the REGOMA trial: a mini-signature of 2 gene transcripts (HIF1A, CDKN1A), 3 miRNAs (miR-3607-3p, miR-301a-3p, miR-93-5p) [26] and phosphorylated acetyl-CoA carboxylase [27] were associated with prolonged survival in recurrent glioblastoma patients treated with regorafenib.

In this real-life study, the methylation status of MGMT was not correlated to the survival supporting its independent role in terms of regorafenib efficacy, as already described in the REGOMA trial.

As reported in the REGOMA trial, the subgroup of patients taking steroids at baseline showed a trend for a shorter survival compared to patients without steroid treatment, despite it not being statistically significant (*p* = 0.08); indeed, the hazard ratio for patients receiving regorafenib and steroid was 0.75 versus 0.34 for patients treated with regorafenib alone. Dexamethasone is an inducer of CYP3A4 and thus it can cause a lower plasma concentration of regorafenib leading to a decrease in its effectiveness. Moreover, corticosteroid is also immunosuppressive and this is another mechanism of interference from steroid [28]. Hence, the dosage of steroid should be limited in patients taking regorafenib.

In our study, regorafenib was found to have a modest toxicity; even if the percentage of patients who developed at least one study drug-related adverse event was high (90.7%), the majority of these were Grade 1–2 adverse events. The most frequent regorafenib grade 3–4 adverse events in our cohort were hand-foot skin reaction, hypertransaminasemia and skin rash, with only one grade 4 skin rash. Overall, 37% of patients required a dose reduction (17% in the Regoma trial), and only three out of 54 patients (6%) required a permanent discontinuation of the drug (7% in the Regoma trial). Regorafenib-induced side effect data should be interpreted with caution due to the retrospective nature of the study; however, in this work the regorafenib dose was reduced in a higher proportion of patients than in the REGOMA trial; it is likely that this may have led to a lower rate of grade 3–4 adverse events.

However, it has been already demonstrated that regorafenib did not affect the patient’s quality of life; indeed, a very recent important study showed that the quality of life does not change during regorafenib treatment and no difference was demonstrated between standard lomustine and regorafenib in terms of quality of life during the REGOMA trial [29].

As aforementioned, since the publication of the Regoma study in January 2019, some papers have been published evaluating the use of regorafenib in small and heterogenous case-series of patients with recurrent high-grade gliomas with disappointing results in terms of efficacy. The first paper, published in February 2019 by Kebir et al. [30], evaluated regorafenib in a small and heterogeneous cohort of six patients, of whom three at the first disease relapsed, while the other three with a number of relapses >2; in addition, 2/6 patients were anaplastic astrocytomas (WHO grade III). In this study, regorafenib achieved a DCR of 0% with an OS less than six months and grade 3 drug-related adverse events in 5/6 treated patients. A further study evaluated the activity of regorafenib in 24 patients with relapsed high-grade glioma [31]; also in said study, the population was very heterogenous with one diffuse midline glioma, one anaplastic oligodendroglioma and one anaplastic astrocytoma other than glioblastoma: moreover, most of the patients were treated over the second relapse; in said study, the median OS after starting regorafenib was 4.1 months and the median PFS was 2.1 months; 13% of patients showed a partial response and a stable disease was demonstrated in another 13% of cases. Likely, the poor efficacy of regorafenib reported in those two studies could be due to the heterogeneity of patients in terms of histology and line of therapy. Conversely, in our retrospective study, we enrolled only patients with a histologically confirmed diagnosis of glioblastoma, and all patients received regorafenib as second-line treatment after the Stupp protocol, making our case series more similar to the Regoma trial.

Several new studies are exploring the possibility of using regorafenib alone or in combination with other drugs in glioblastoma patients. Firstly, an observational “real-world”, prospective study is ongoing in Italy to analyze the role of regorafenib in recurrent glioblastoma outside clinical trials (REGOMA-Oss). Secondly, a phase II study (NCT04051606) is evaluating the use of regorafenib in bevacizumab refractory glioblastoma or other WHO grade IV glioma patients (gliosarcoma or small cell glioblastoma). The primary endpoint of said study is the overall survival. Thirdly, some ongoing studies are also evaluating the hypothesis of using regorafenib in newly diagnosed patients with glioblastoma, after surgery and concomitant chemoradiotherapy with temozolomide. GBM AGILE (NCT03970447) is an international, seamless Phase II/III response adaptive randomization platform trial designed to evaluate multiple therapies in newly diagnosed and recurrent glioblastoma, in which, regorafenib is proposed as first-line treatment in patients with newly diagnosed MGMT unmethylated glioblastoma, after concomitant chemoradiotherapy treatment with temozolomide, or as treatment at the time of the first relapse in recurrent glioblastoma patients, regardless of the MGMT methylation status. Fourthly, a phase I study analyzing the safety and pharmacokinetics of regorafenib in combination with temozolomide and radiotherapy in newly diagnosed glioblastoma patients (REGOMA 2) will be soon launched in Italy. Fifthly, since it has been demonstrated that the action of regorafenib on the tumor microenvironment by a reduction of immunosuppressive myeloid cells may lead to a greater efficacy of immune checkpoint inhibitors (ICI) [16], a new basket trial is ongoing (NCT04704154) which evaluates the combination of regorafenib plus nivolumab in many types of cancer, including recurrent glioblastoma.

## 5. Conclusions

In conclusion, we performed a real-life study analyzing the activity and safety of regorafenib in recurrent glioblastoma patients when used as second-line treatment. Although further prospective and randomized studies will be necessary to confirm the role of regorafenib in glioblastoma patients, our results were superimposable to the ones reported in the REGOMA trial.

## Figures and Tables

**Table 1:** Characteristics of the enrolled patients.

Characteristics	Total (%)
No. of patients	54 (100)
Median Age (range)	55 (30–77)
Gender	
Male	38/54 (70.4)
Female	16/54 (29.6)
Type of first surgery	
Radical surgery	28/54 (52)
Partial surgery	26/54 (48)
Stupp protocol completed	
yes	35/54 (65)
no	19/54 (35)
Median time from diagnosis to regorafenib therapy	14.3 months
ECOG performance status	
0–1	54 (100)
Surgery at time of recurrence	16/54 (29.6)
Corticosteroid use	
yes	22/54 (40.7)
no	32/54 (59.3)
IDH mutation status	
wild-type	49/54 (90.7)
mutated	5/54 (9.3)
MGMT methylation status	
methylated	28/53 (52.8)
unmethylated	25/53 (47.2)
Third-line therapy	
yes	35/54 (65)
no	19/54 (25)

**Table 2:** Univariate analysis for overall survival. Met = methylated; unmet = unmethylated; wt = wild-type; mut = mutated.

Variable		OS		
		Median (Months)	95% CI	*p*
MGMT Status				0.20
	Met Unmet	10.2 7.6	4.5–15.8 3.7–11.6	
IDH status				0.37
	wt mut	10.2 Not reached	6.9–14.4	
Age				0.17
	≤65 years >65 years	12.03 7.4	6.6–17.4 5.9–8.9	
Second surgery				0.3
	yes	12.9	4.4–21.4	
	no	8.6	4.7–12.5	
Steroid at baseline				0.09
	yes	7.4	4.7–10.2	
	no	12.9	9.2–16.6	
Third Line therapy				0.8
	yes no	10.2 8.0	6.3–14.02 0.01–16.9	

**Table 3:** Multivariate analysis for overall survival.

Variables	OS	
	HR (95% CI)	*p*
MGMT status (unmet vs. met)	1.4 (0.6–3.05)	0.3
Age (≤65 vs. >65)	0.4 (0.1–1.2)	0.1
Steroid at Baseline (no vs. yes)	0.4 (0.2–1.06)	0.07

**Table 4:** Neuro-radiological assessment according to RANO criteria. Bold and Italics: the sum of the prior characteristics.

Overall Responses according to RANO Criteria	No. (%)
Complete Response	0 (0)
Partial Response	4 (7.4)
***Objective Response Rate***	***4 (7.4)***
Stable Disease	21 (38.9)
***Disease Control rate***	***25 (46.3)***
Progressive Disease	29 (53.7)

**Table 5:** Drug-related adverse events according to the CTCAE v 5.0.

Adverse Events	Grade 1	Grade 2	Grade 3	Grade 4
Hand and Foot skin reaction	17 (31%)	3 (5%)	4 (7%)	
Hypertension	7 (13%)	3 (5%)		
Fatigue	17 (31%)	7 (13%)	1 (2%)	
Mucositis	8 (15%)	5 (9%)	1 (2%)	
Dysphonia	5 (9%)			
Fever	10 (18%)	4 (7%)		
Diarrhea	6 (11%)	1 (2%)		
Blood Bilirubin Increased	8 (15%)	3 (5%)		
Hypertransaminasaemia	5 (9%)	1 (2%)	2 (4%)	
Serum Amylase/Lipase Increased	1 (2%)		1 (2%)	
Skin Rash	5 (9%)	4 (5%)	3 (5%)	1 (2%)
Thrombocytopenia	5 (9%)	2 (4%)		
Hypothyroidism	2 (4%)	1 (2%)		

## Data Availability

The data were not publicly archived.
